# Evolution process and failure mechanism of a large expressway roadside landslide

**DOI:** 10.1038/s41598-023-32055-z

**Published:** 2023-03-24

**Authors:** Jian Zhang, Shihua Zhang, Yong Ding, David Z. Zhu

**Affiliations:** 1 School of Civil and Environmental Engineering, Ningbo University, Ningbo, 315211 China; 2 Shenzhen Geotechnical Investigation & Surveying Institute (Group) Co. Ltd., Shenzhen, 518028 China; 3 Department of Civil and Environmental Engineering, University of Alberta, Edmonton, AB T6G 2W2 Canada

**Keywords:** Natural hazards, Civil engineering

## Abstract

Site investigation, deformation monitoring, laboratory test, and theoretical calculations were used to analyze the evolution details of a large expressway roadside landslide during the start-up sliding process. The monitoring results show that the initial deformation and failure occurred on the protective wall at the slope toe, then gradually developed to the upper part of the slope, and finally led to tensile cracks at the slope trailing edge. Accelerated deformation of the slope support structures, such as the protective wall at the slope toe, the anti-slide pile, and the anchor cable, were observed during the continuous extreme rainfall. The infiltrated rainwater can change the weight, the osmotic pressure, the anti-sliding force, the sliding force of the sliding mass, and further soften the fully weathered tuff soil and reduce its strength, resulting in the landslide occurrence. Block the slope surface runoff is an effective measure to reduce the landslide risk. The current analysis will be helpful to the prevention, control, and emergency disposal of similar landslides.

## Introduction

With the development of society and economy, large-scale engineering constructions have demonstrated the ability of human beings to conquer nature, but such activities have also brought some negative effects, such as large-scale landslides caused by the construction of highways, railways and other infrastructure due to the excavation of the foothills^1–4^. The construction of infrastructure such as highways and railways is an inevitable product of the development of human society and an indispensable part of human life. Therefore, disasters (such as engineering landslides) caused by engineering constructions are also inevitable^5–7^. The slopes constructed by engineering constructions have been reinforced normally but may still have bad consequences under complex geological or extreme weather conditions^8,9^. In addition, the frequency of extreme weather (storms, typhoons) has increased significantly in recent years^10,11^, and the pressure of human beings on various natural disasters has become more and more prominent. Studying the development characteristics and occurrence mechanism of various landslides is of great significance in order to deal with this kind of disasters. Finding disaster risks well in advance protection can reduce casualties and property losses.

Early detection of disaster risk is one of the most effective strategies to deal with various large-scale natural disasters. Landslide risk early warning represented by rainfall monitoring, soil water content and pore water pressure monitoring and deformation monitoring is widely used in engineering^12–18^. The main indicators to identify landslide risk include the abnormal increase of rainfall^19–21^, the increase of soil moisture content^13,22–27^, the acceleration of slope deformation^28,29^. The common feature of these monitoring indicators is “abnormal change”. The focus of slope monitoring and early warning is to capture the early slip characteristics. Landslide initiation process is a key stage in understanding the occurrence and development of landslides, which provides a direct information source for understanding the occurrence mechanism and triggering factors of landslides. For example, Keefer et al.^30^ stated that analysis of the characteristics for the landslide initiation stage can preliminarily judge the depth and scope of the landslide. De Vita et al.^31^ evaluated landslide risk by estimating the hydraulic thresholds in the landslide initiation stage. Lainas et al.^32^ assessed the slope slip risk by identifying rainfall thresholds that trigger the landslide initiation. Cogan et al.^33^ use physical experiments to evaluate the relationship between physical factors such as rainfall, slope inclination and landslide initiation. The above studies show that the trigger factors and the slope change characteristics in the landslide initiation stage have a close relationship with the further development of the slope sliding. Therefore, the identification and analysis of the deformation characteristics of the slope during the start-up sliding process are beneficial to the early recognize of the landslide risk.

In this study, a combination of site investigation, deformation monitoring, laboratory test and theoretical calculation is used to analyze the evolution details of a large expressway roadside landslide during the start-up sliding process (from May 2018). Detailed geological investigation on the landslide region is conducted. The landslide is described, and hydraulic conditions and weather conditions in the landslide region are analyzed. Typical events of the landslide during the evolution process from May 2017 to September 2018 are recorded. The slope deformation characteristics at different time nodes, corresponding to those typical events and the emergency treatment and drainage measures during extreme rainfall, are analyzed in detail. The depth and position of the slip surface determined based on the statistical method of borehole exploration are displayed. Based on the in-situ data obtained by monitoring technology, such as clinometer results, slope toe protective wall and anti-slide pile deformation, anchor cable tension change and groundwater change, the start-up sliding characteristics and the influence of heavy rainfall on slope deformation are analyzed. The laboratory test results of soil samples taken from the field and the theoretical calculation data of slope stability are used to directly reflect the influence of heavy rainfall on slope stability. Finally, the occurrence mechanism is analyzed according to the obtained field data.

## Materials

### Regional geological background

**Figure 1:**
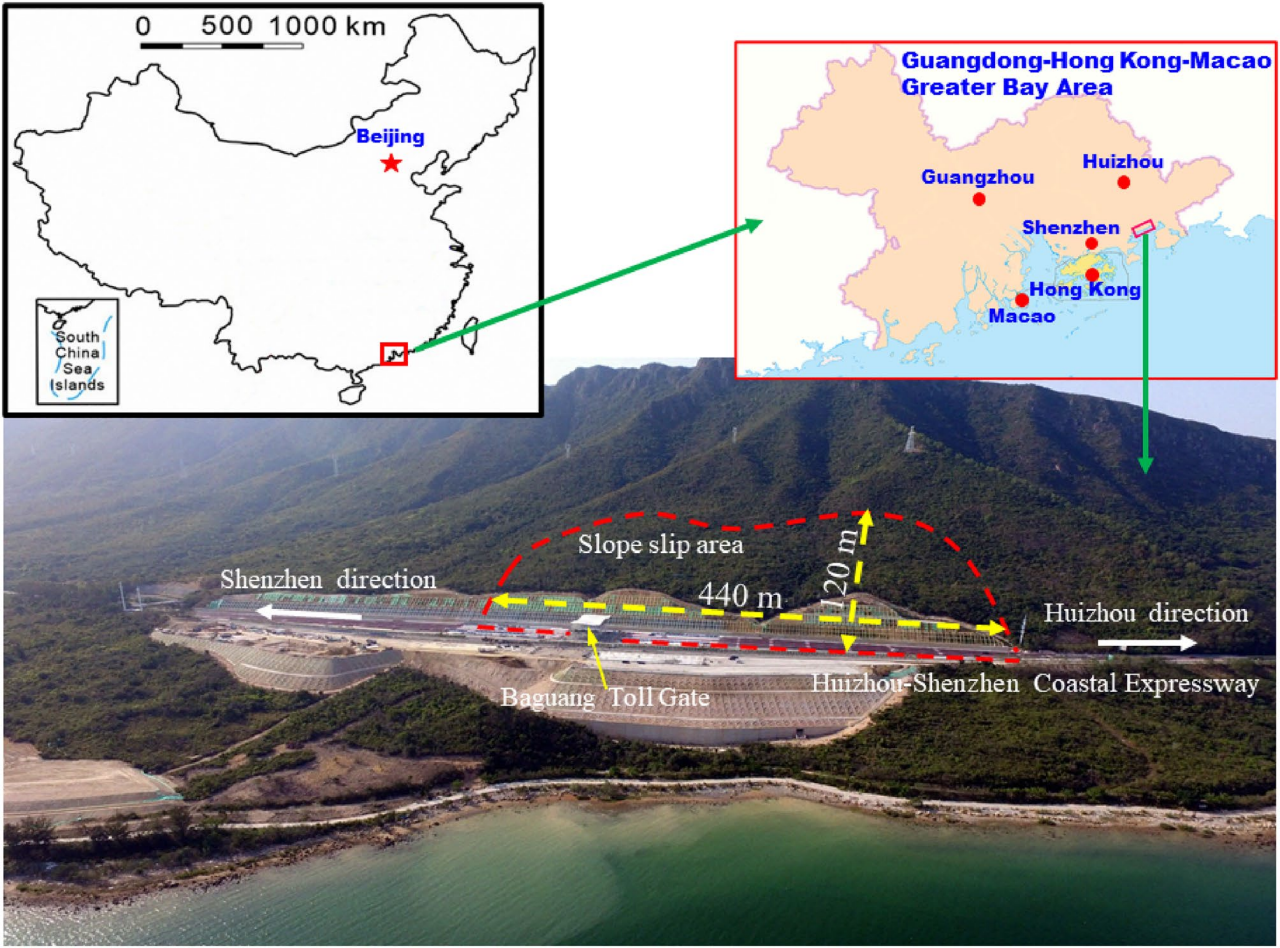
The location of the landslide, in the Guangdong-Hong Kong-Macao Greater Bay Area, South of China (the area maps in the figure above were created based on the editable EPS original file (http://bzdt.ch.mnr.gov.cn/index.html) and software Adobe Illustrator CC 21.0.0 (64-bit)).

The location of the landslide is in the Guangdong-Hong Kong-Macao Greater Bay Area, Baguang toll gate of Huizhou-Shenzhen Coastal Expressway (Fig. 1). Mountain excavation is required on the north side of the expressway due to engineering construction. The maximum slope cutting height is about 32 m. The total length of the slope is about 675 m, the slope height is from 15 to 32 m, and the slope inclination is 45° to 50°. There are no obvious fault structures and other typical geological structures at the slope region. The slope area is close to the bay (Shenzhen Baisha Bay), and its location is high in the north and low in the south, with a slope in the range of 20° to 40°. The expressway at the foot of the slope crosses the hillside from the west to east.

From the top to bottom, the stratum can be divided into: Quaternary artificial filling layer (Q^ml^), Quaternary proluvial slope (Q_4_^dl+pl^) cohesive soil boulders, Quaternary residual (Q_2_^el^) silty clay, and the tuff of Jurassic Wutongshan Formation (J_2–3_w). The lithology of each stratum is artificial filling, boulder containing cohesive soil, silty clay, and completely weathered siltstone (Fig. 3). Among them, artificial fill has loose structure and good water permeability. The soil layers, e.g., silty clay containing breccia, silty clay, and completely weathered tuff, have poor water permeability. The Quaternary artificial filling layer, slope diluvium, eluvium and completely weathered layer are all water bearing rock formations.

The bedrock is tuff with obvious weathering stratification. There is fissure water in the weathered fissure zone. Distribution of fissure water has obvious anisotropic characteristics. In the section with relatively developed joint fissures, the fissure water is relatively rich and has strong water permeability. Bedrock fissure water is mainly supplied by vertical infiltration and lateral runoff. The overall water volume is small and uneven, with micro pressure bearing.

### Landslide description

**Figure 2:**
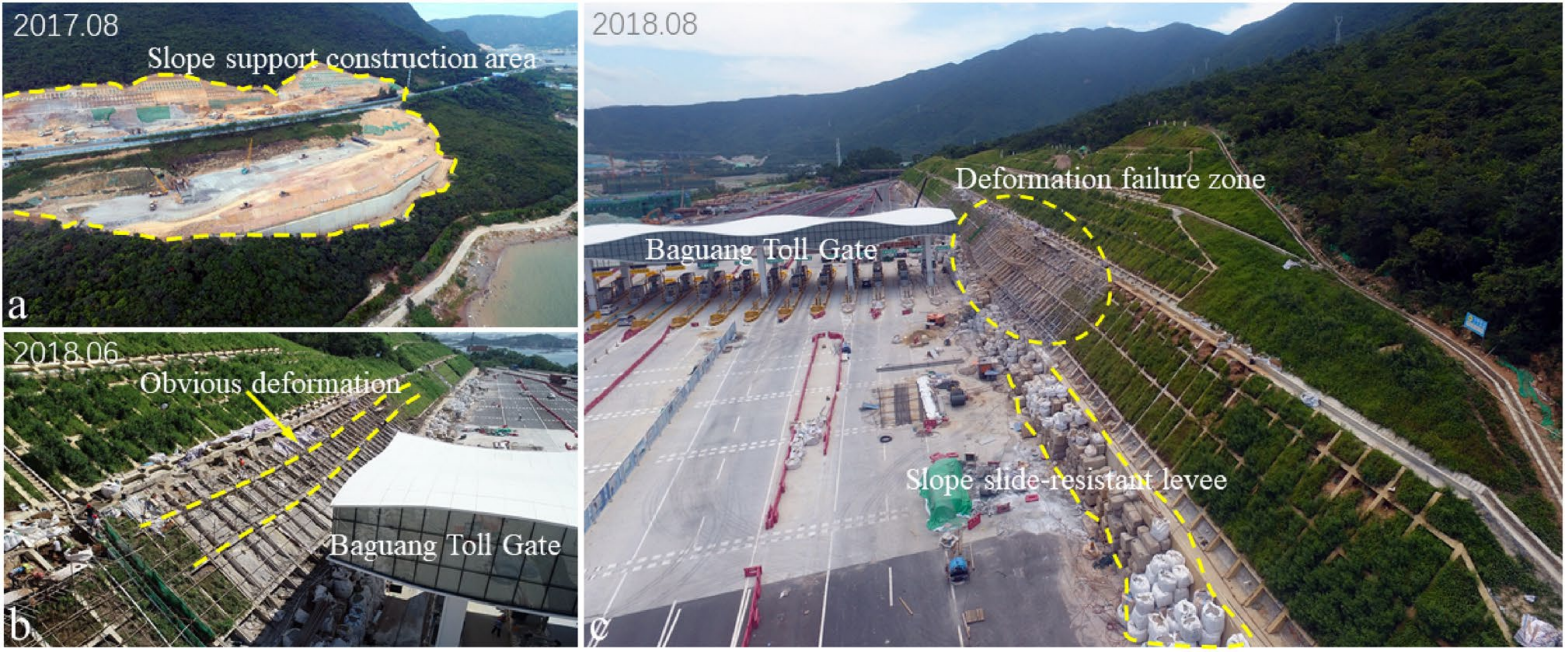
Typical stages before the formation of the Baguang Toll Landslide: (**a**) excavation of the Baguang toll station slope in the initial construction stage; (**b**) large deformation threatening the toll station safety; (**c**) slope supporting structure appears obvious deformation and failure area for suffering typhoon.

The excavation and construction of the Baguang toll gate slope started in August 2017 (Fig. 2a). It is a typical landslide caused by extreme rainfall and engineering construction (excavation of the mountain foot). The construction of this cutting slope was completed in January 2018, and obvious deformation was observed since May 2018. After two heavy rains (June 7 and August 28, 2018), the slope deformation was accelerated, which threatened the overall safety of the expressway and the toll station (Fig. 2b,c). On June 7, 2018, due to the influence of Typhoon “Aiyuni”, the maximum daily rainfall intensity reached 159 mm/day, and the cumulative rainfall in three days reached 489.3 mm. On August 28, 2018, a continuous extremely heavy rainstorm occurred, lasting about a week. The peak rainfall intensity reached 112.4 mm/h, the maximum daily rainfall reached 417.2 mm/day, and the cumulative rainfall reached 1926.4 mm. The field survey results show that: the entire landslide region was about 440 m long and 120 m wide (Fig. 1), with the area about 48,400 m^2^. The bottom sliding surface developed along the weak structural plane (mainly completely weathered tuff). The thickness of the landslide mass was uneven, with an average thickness of about 20 m. The total landslide volume was about 6.8 × 10^4^ m^3^. The main sliding direction was about SE 151°. Owning to the above reasons, Baguang Toll Landslide can be categorized as a large landslide with a middle-level depth. Based on the “Cruden and Varnes’ classification”^34^, typical category of this slope failure is “Translational sliding”.

**Figure 3:**
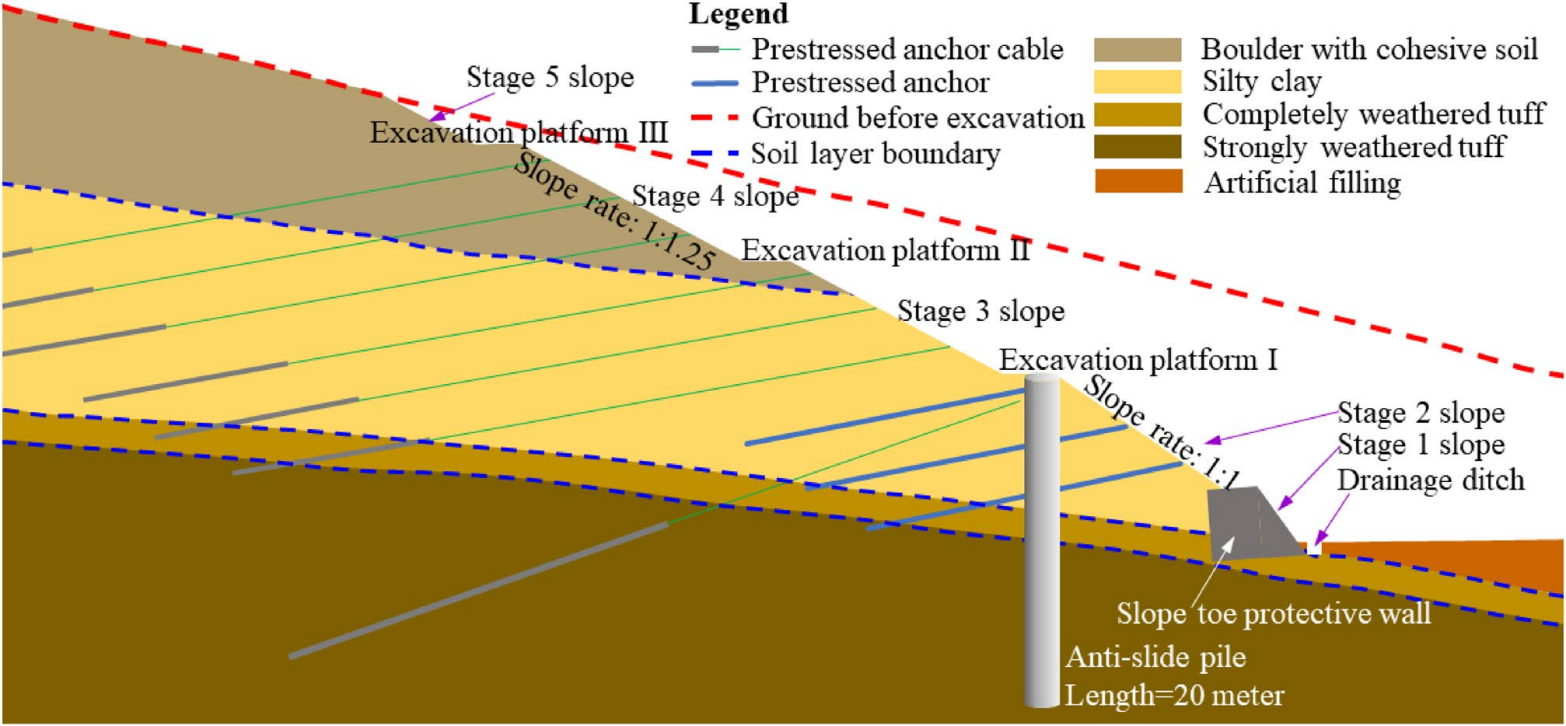
Typical geological cross-section and support measures during the slope construction.

Slope mileage of the Baguang toll gate is K28 + 220.0 to K28 + 885.0 (K28 is the section number of the Huizhou-Shenzhen Coastal Expressway), with a total length of 665 m. Maximum excavation height during slope construction is 32.2 m. Typical geological cross-section of the slope (with the maximum excavation height) and slope support mode during the construction stage is shown in Fig. 3. Five stage slopes are set at the maximum excavation height to form a relatively stable initial state. Stage 1 slope uses the declining slope toe protective wall, stage 2 slope uses the anchor lattice beam and anti-slide pile, stage 3 and 4 slopes use the anchor lattice beam, and stage 5 slope uses the mesh planting protective means. Excavation platform was set between each stage slope for setting drainage ditches and daily safety patrol. The slope is mainly composed of four soil layers in natural state, i.e., boulder with cohesive soil, silty clay, completely weathered tuff, and strongly weathered tuff. The landslide sliding surface is mainly developed in the completely weathered tuff soil layer.

### Hydrogeological setting

No obvious surface water was found in the slope region. Groundwater mainly exists in the overlying soil layer and the cracks of the weathered bedrock. Groundwater is mainly supplied by atmospheric precipitation and lateral seepage of pores and cracks between rock and soil layers. Groundwater is mainly discharged by lateral runoff. Affected by terrain, groundwater moves from north to south. The groundwater level can fluctuate with season and rainfall, and the water level can increase significantly in the rainy season. The field borehole survey found that groundwater was exposed in some boreholes. During the survey, the buried depth of the site borehole water level was measured to be 1.0 to 9.6 m, and it indicates that the annual variation range of the groundwater level is about 1.0 to 5.0 m.

The recharge of groundwater depends on atmospheric precipitation and lateral seepage of pores and cracks in rock and soil layers. The discharge of groundwater is mainly lateral runoff. Groundwater fluctuation has a significant impact on slope stability. When encountering continuous heavy rainfall, groundwater will rise sharply. Due to the poor permeability of the completely weathered tuff soil, the rock and soil will be partially saturated, which will increase the bulk density and reduce the soil shear strength.

### Weather conditions

**Figure 4:**
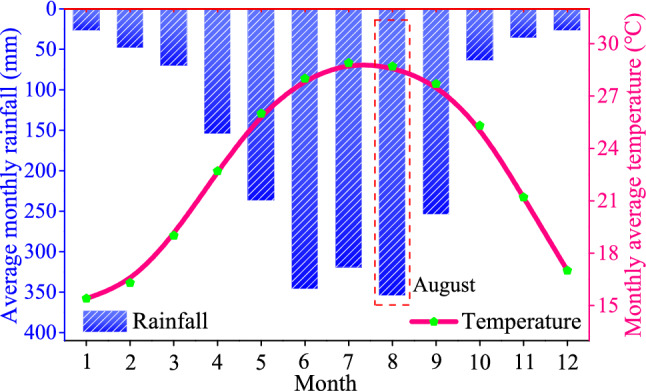
Monthly average rainfalls and monthly average temperature in 34 years of the landslide region (from 1981 to 2014).

The landslide region is in a subtropical monsoon climate with a long summer and a short winter and abundant rainfall, where natural disasters can frequently occur^35,36^. The average monthly rainfall and temperature (from 1981 to 2014) distributions of each month are shown in Fig. 4. The annual average temperature is 23.0 ℃, the extreme historical maximum temperature is 38.7 ℃, and the historical extreme minimum temperature is 0.2 ℃. In a year, the average temperature in January is the lowest, with an average of 15.4 ℃, and the average temperature in July is the highest, with an average of 28.9 ℃. Average annual precipitation is 1935.8 mm, and 86% of the annual rainfall occurs in the rainy season (from April to September). Summer lasts for more than 6 months (an average of 196 days).

The total annual rainfall in 30 years is 1966.3 mm. The rainy season is from April to September every year, and most of them are typhoon rainstorms. Maximum annual precipitation is 2747 mm (2001), and the minimum annual precipitation is 912.5 mm (1963). The annual maximum continuous rainfall is 459.3 mm, the maximum daily average rainstorm is 282 mm (June 2008), and the maximum hourly rainfall is 114.8 mm/h (March 2014). The average annual rainfall days are 144.7 days, and the longest continuous rainfall days are 20 days.

## Methods

### Site investigation

**Figure 5:**
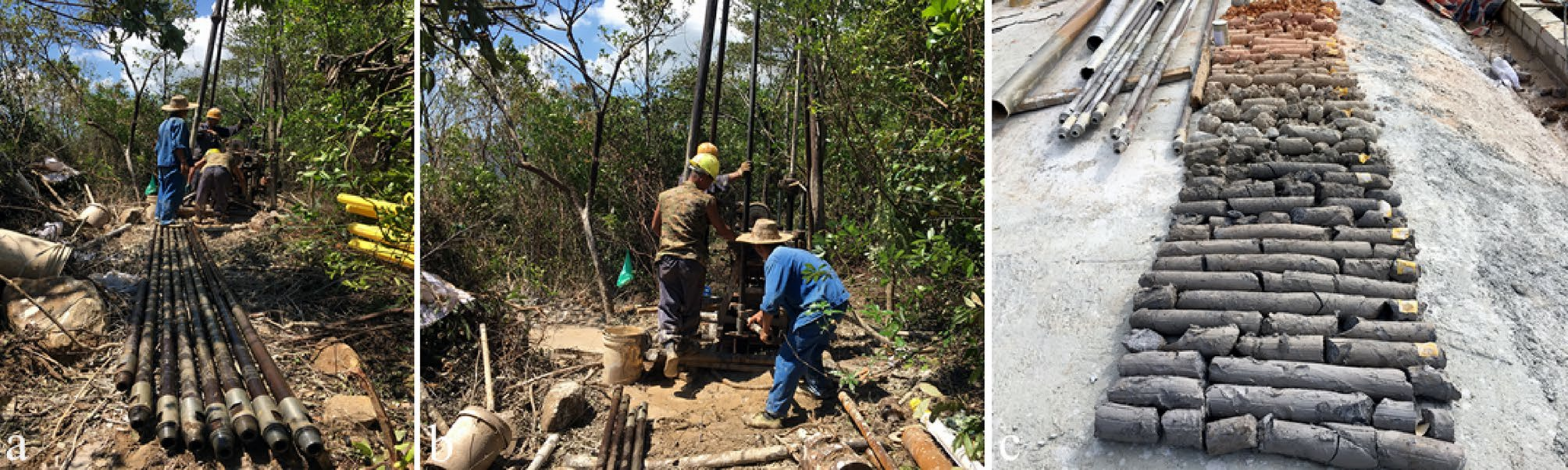
Drilling detection construction processes: (**a,b**) Drilling process and drill pipe installation; (**c**) complete core sample taken from borehole.

Through field survey and observation, the deformation and failure evolution trend of the slope was identified. Starting from the slope support construction, dynamic observation and patrol inspection were carried out for the slope safety after the triggering events such as engineering construction and rainfall. Key observation indicators include visible cracks, integrity of structural facilities (e.g., protective wall, frame beams, anti-slide pile), drainage conditions and integrity of drainage facilities (e.g., drainage ditch, catchment facilities). The observation results were stored in the form of on-site photos and videos and formed into on-site data database. Through the observation data, the existing slope state can be evaluated in real time, the stability evolution characteristics of the slope can be found, the possible situation of the slope can be predicted, and the engineering means can be adjusted in time to ensure the construction safety. To obtain the slope sliding surface depth and the physical parameters of the rock, soil, and slope structure, drilling sampling was carried out at different parts of the slope. The drilling process is shown in Fig. 5. Based on the core samples taken from the borehole, the physical parameters of the soil and rock can be further obtained, and the slope slip surface position can be located.

### Deformation monitoring

**Figure 6:**
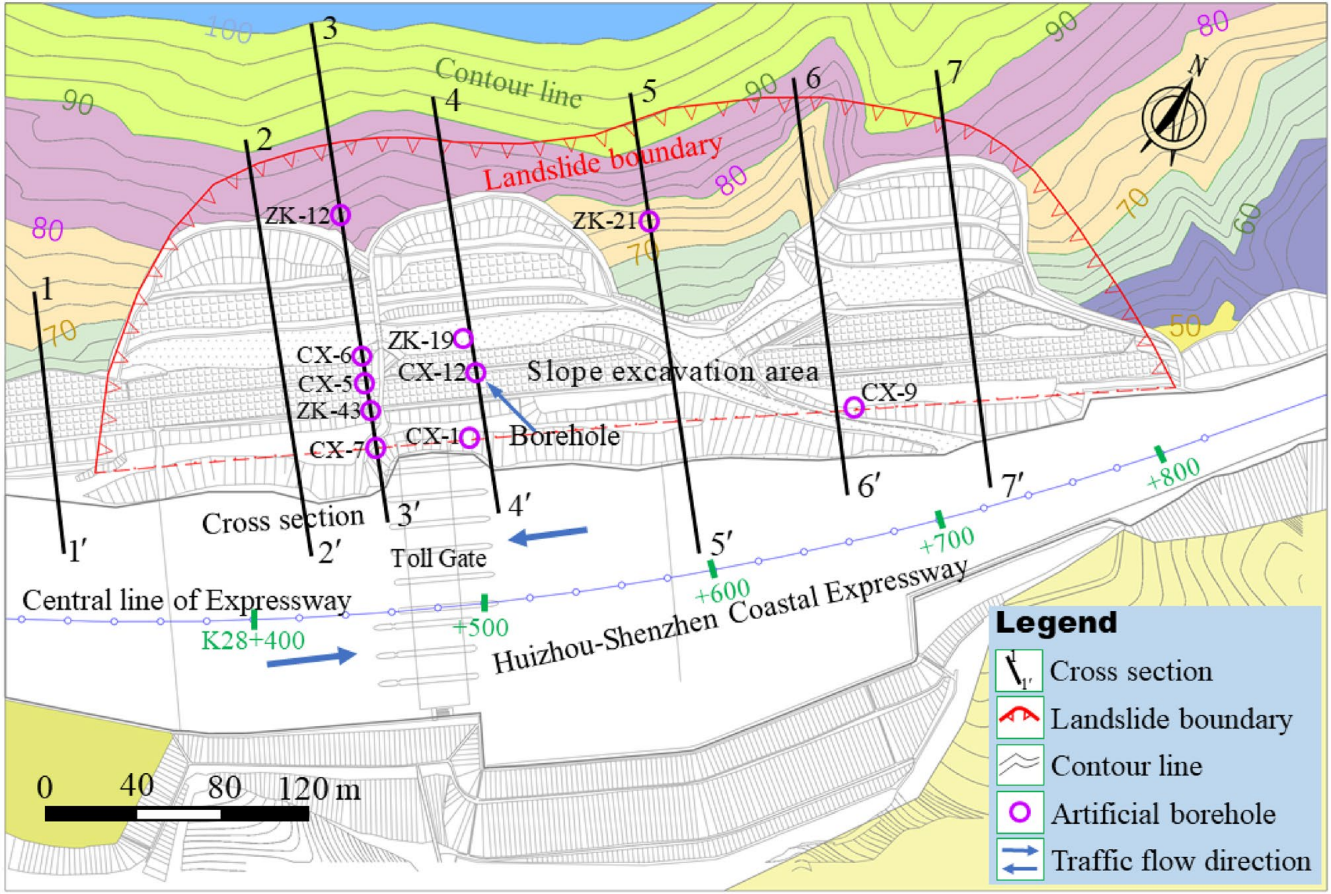
The distribution of the observation cross sections and part of boreholes.

Deformation monitoring is an important means to ensure the construction safety^5,37^. The monitoring data are also an index to evaluate the effectiveness of reinforcement measures. Deformation monitoring tools used in this slope engineering include inclinometer, automatic total station. The inclinometer was used to obtain the deformation characteristics of the deep part of the slope, which can determine the sliding surface location. The theodolite was mainly used to capture the micro deformation of the slope surface. To obtain systematic observation data, several cross sections were arranged in the slope area. The cross sections and part of artificial boreholes are shown in Fig. 6. Cross sections are mainly distributed inside and around the slope construction and excavation area. The total number of boreholes in this slope engineering exceeds 50, and only a number of boreholes are marked in Fig. 6.

**Figure 7:**
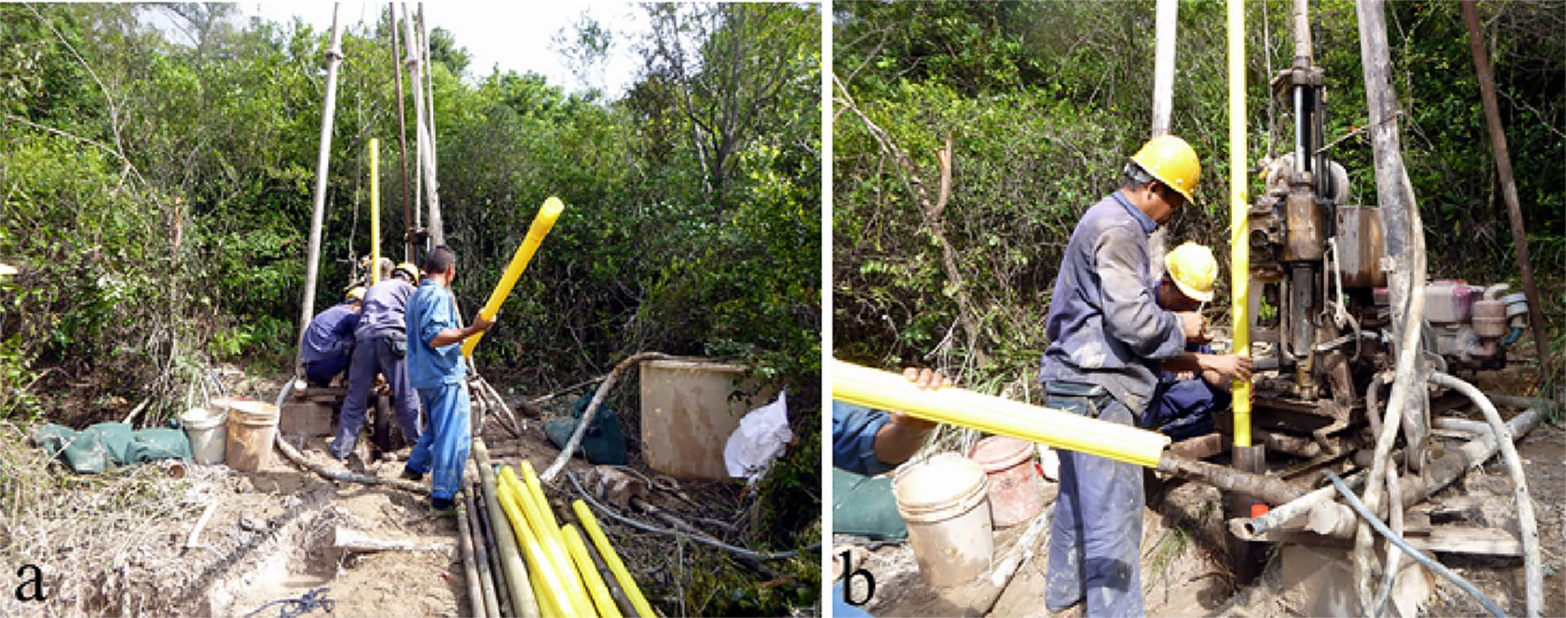
Installation of inclinometer pipe.

Geological drilling can be used to obtain the rock and soil parameters. Artificial boreholes can be used for the groundwater observation and a special position for inclinometer. When the inclinometer is embedded in the borehole, the deep displacement of soil can be continuously monitored. Figure 7 shows the scenario of burying inclinometer pipe on site. The result of inclinometer monitoring is important to judge the slope state and determining the slip surface depth.

**Figure 8:**
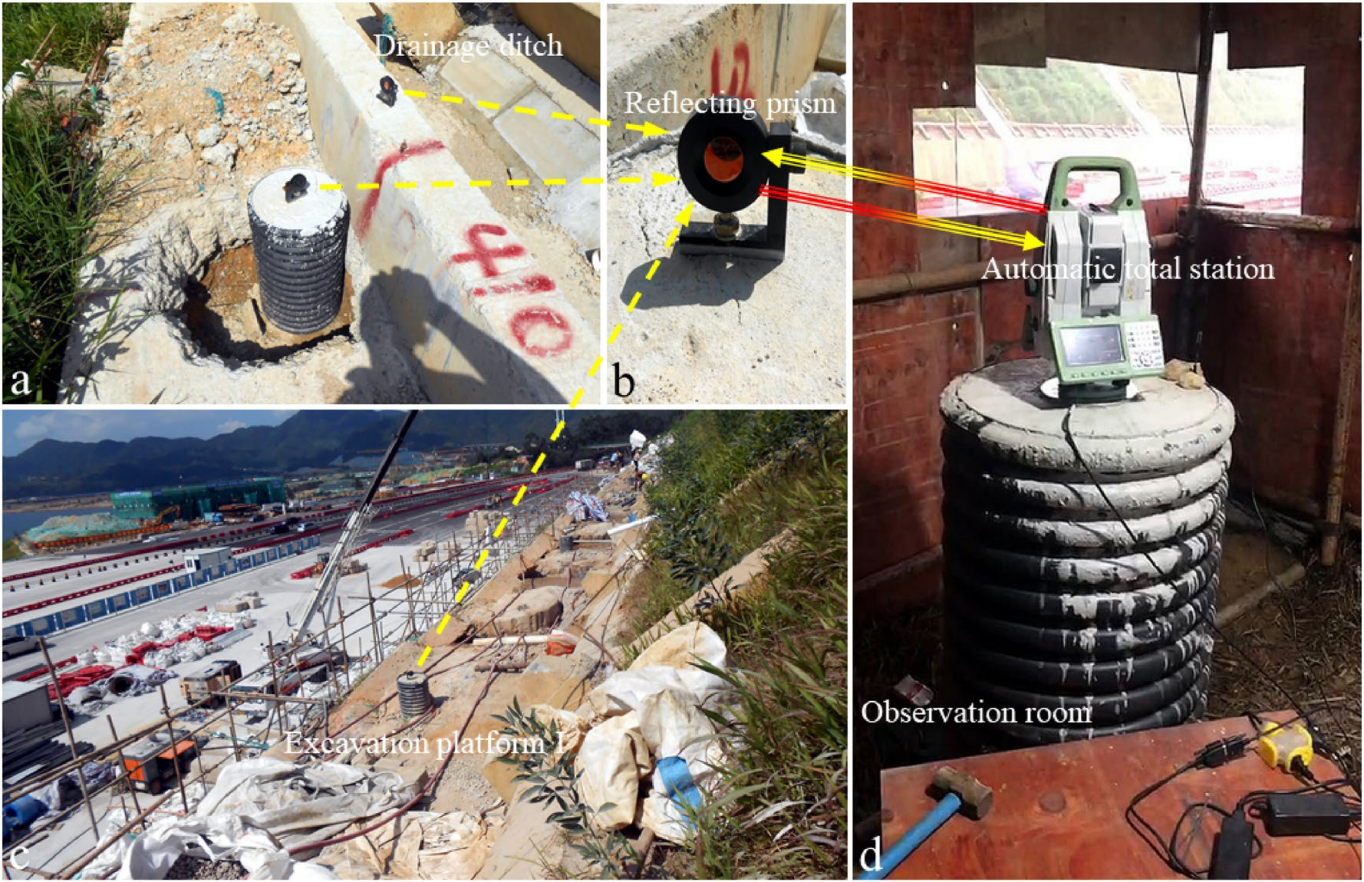
Automatic total station deformation monitoring system. (**a–c**) Show the prisms placed in the field; (**d**) shows the automatic total station placed in the observation room.

The deformation monitoring system composed of electronic theodolite and optical prism can quantify the deformation of the slope surface and slope support structures. The monitoring results can be used to evaluate the deformation characteristics of slope support structures (such as slope toe protective wall, anti-slide pile, frame beam). The field layout of automatic total station displacement monitoring system is shown in Fig. 8. Daily statistics can be made on the slope deformation within 2 km around the observation room. This system can better capture the accelerated deformation characteristics of slope after encountering heavy rainfall.

### Laboratory test

To obtain the physical and mechanical parameters of slope soil, laboratory tests were carried out on the soil samples collected on site. The main physical tests include basic physical index tests (including water content, density, bulk density, void ratio, liquid limit, plastic limit) and mechanical parameter tests (including cohesion, internal friction angle). Test standards are based on the national standard (GB/T 6003.1-2012) of People’s Republic of China. The test results can not only be used to understand the distribution characteristics of physical and mechanical parameters of the slope soil in natural state, but also provide calculation inputs for the slope stability analysis.

### Theoretical calculation

The unbalanced thrust transfer coefficient method (refer to the national standard (GB50330-2013) of People’s Republic of China) was used to calculate the slope safety factor under different conditions. In this method, the slope sliding surface is assumed to be a broken line, and the sliding mass is divided into strips. The iterative calculation is carried out according to the stress of each strip until the slope safety coefficient is solved. The slope factor of safety (FS) calculation equation is expressed as follows:

1 $$FS = \frac{{\sum_{i = 1}^{n - 1} {(R_{i} \prod\limits_{j = i}^{n - 1} {\psi_{j} ) + R_{n} } } }}{{\sum\limits_{i = 1}^{n - 1} {(T_{i} \prod\limits_{j = i}^{n - 1} {\psi_{j} ) + T_{n} } } }},$$

where,

2 $$\psi_{j} = \cos (\theta_{i} - \theta_{i + 1} ) - \sin (\theta_{i} - \theta_{i + 1} )\tan \phi_{i + 1} ,$$

3 $$\prod_{j = i}^{n - 1} {\psi_{j} = \psi_{i} \cdot \psi_{i + 1} \cdot \psi_{i + 2} \cdots \cdot \psi_{n - 1} } ,$$

4 $$R_{i} = N_{i} \tan \phi_{i} + c_{i} l_{i} ,$$

5 $$T_{i} = W_{i} \sin \theta_{i} + P_{Wi} \cos \left( {\alpha_{i} - \theta_{i} } \right),$$

6 $$N_{i} = W_{i} \cos \theta_{i} + P_{Wi} \sin \left( {\alpha_{i} - \theta_{i} } \right),$$

7 $$W_{i} = V_{iu} \gamma + V_{id} \gamma^{\prime},$$

where, $\psi_{i}$ is the transfer coefficient, $R_{i}$ (kN/m) is the anti-sliding force of the strip *i* (sliding mass element for calculation), $T_{i}$ (kN/m) is the sliding force of the strip *i*, $N_{i}$ (kN/m) is the reaction force of the strip *i* (on the normal of the sliding surface), $c_{i}$ (kPa) is the cohesive force of the rock and soil on sliding surface, $\varphi_{i}$ (°) is the internal friction angle, $l_{i}$ (m) is the bottom length of the strip *i*, $\alpha_{i}$ (°) is the average angle value between water table and horizontal plane of the strip *i*, $W_{i}$ (kN/m) is the weight of the strip *i*, $\theta_{i}$(°) is the angle between the bottom of strip *i* and the horizontal, $P_{Wi}$ (kN/m) is the osmotic pressure of the strip *i*, $V_{iu}$ (m^3^/m) is the volume above phreatic line of the strip *i*, $V_{id}$ (m^3^/m) is the volume below phreatic line, $\gamma$ (kN/m^3^) is the natural bulk density of the rock or soil, $\gamma^{\prime}$ (kN/m^3^) is the floating bulk density of rock or soil, $\gamma_{sat}$ (kN/m^3^) is the saturated bulk density of rock or soil.

Based on the above calculation equations, the slope FS can be solved by the following methods: (1) Determine the slip surface position based on the on-site drilling and inclination results; (2) Set division number of the sliding mass; (3) Take the groundwater level, rainfall intensity as the boundary conditions to obtain the calculation results (FS) under different conditions.

## Results

### Characteristics of slope starting slip

For the expressway construction, excavation engineering of the natural slope at the location of Baguang toll gate began in May 2017. Construction of Baguang toll gate began in August 2017 and was completed by January 2018. During the construction of the toll gate and roadside slope support, there was hardly any rainfall.

**Figure 9:**
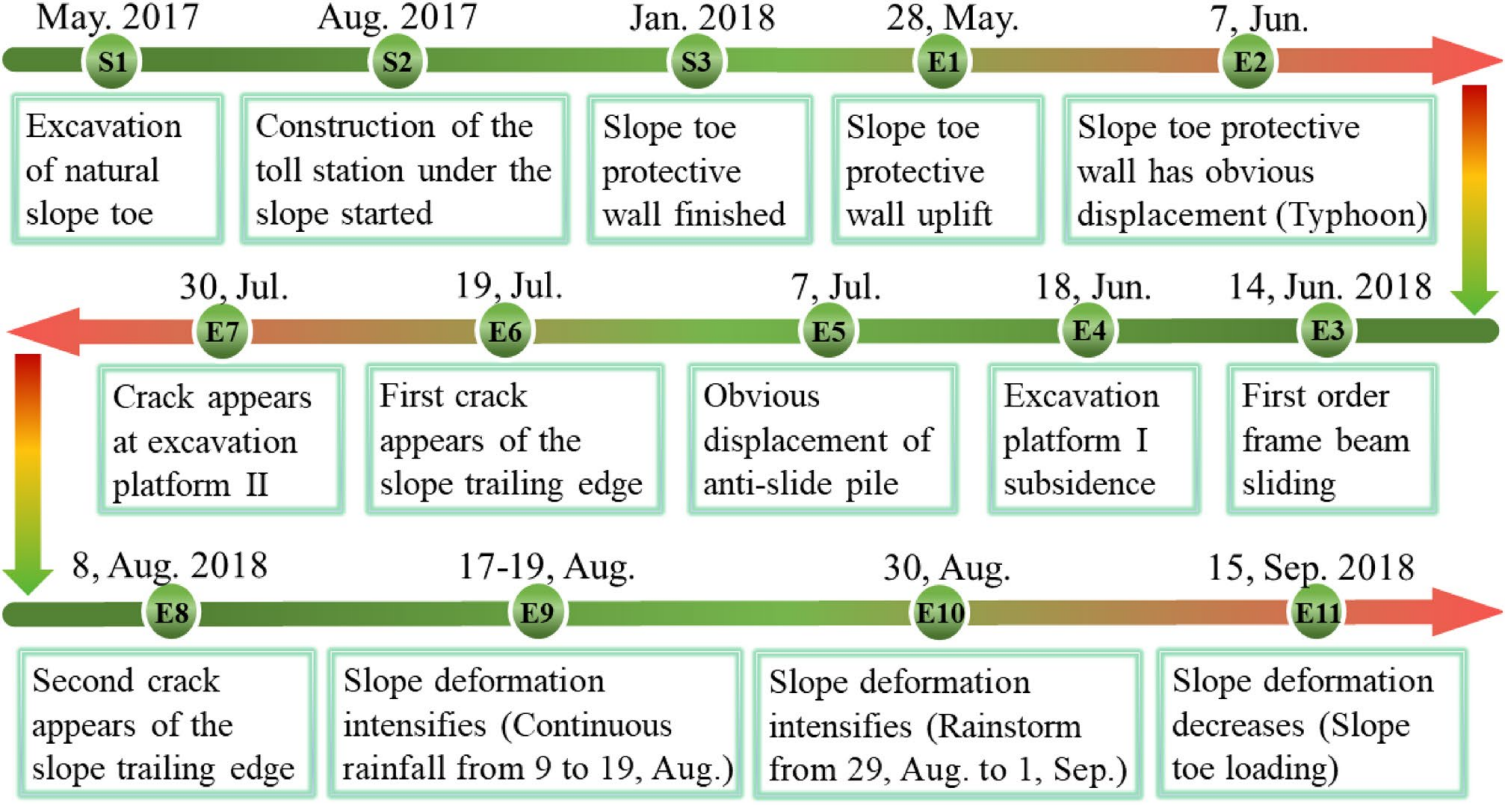
Evolution process and typical events timeline of the Baguang toll gate landslide from May 2017 to September 2018 (S1, S2, S3 represents the time nodes in the construction process, E1-E11 represents the time node when the slope gradually tends to instability after construction).

**Figure 10:**
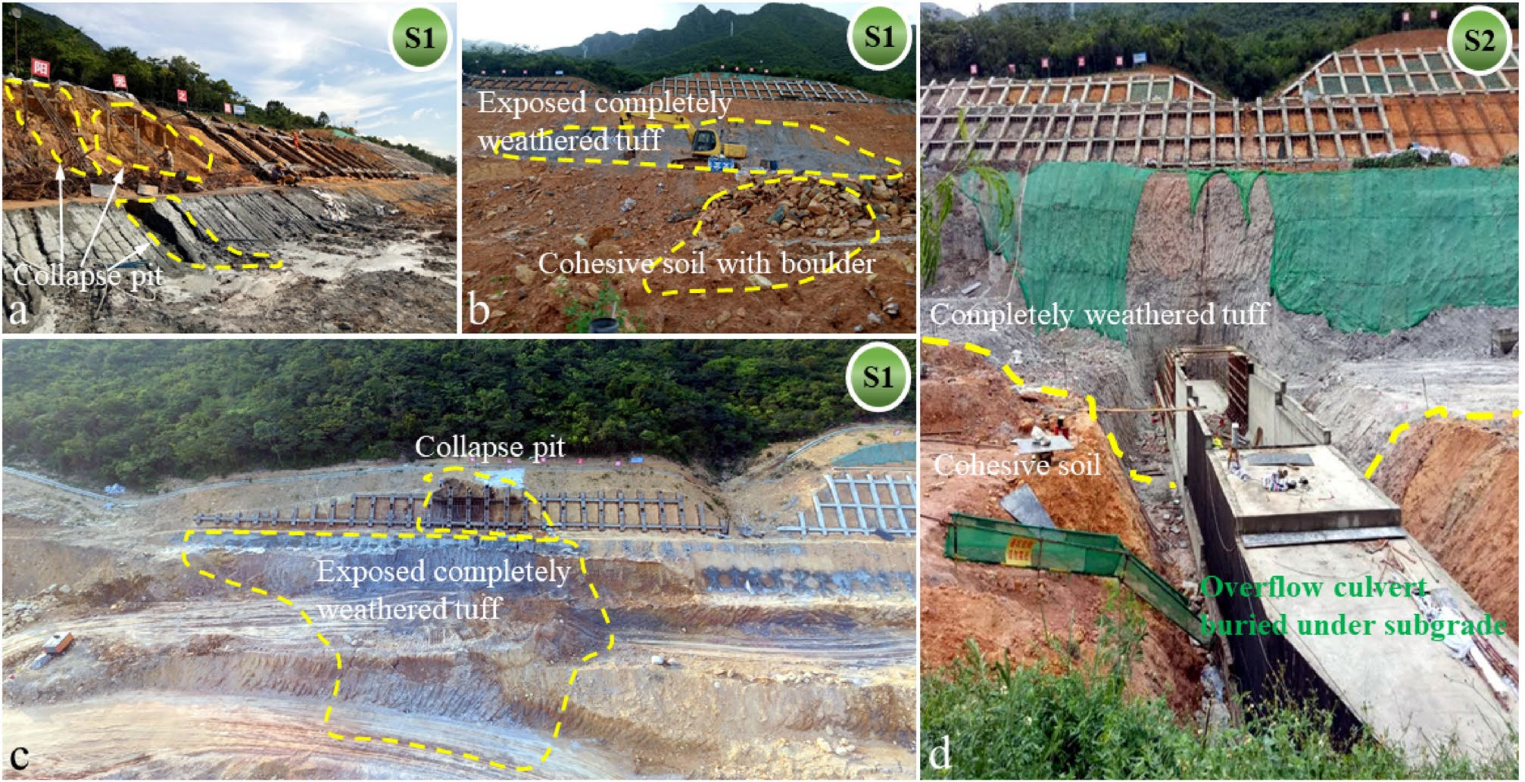
Excavation during construction of the roadside slope and toll gate (S1 and S2 correspond to the time nodes in Fig. 9). (**a**) Collapse pit caused by roadside slope excavation. (**b**) Distribution of weathered tuff on roadside slope and cohesive soil containing boulder. (**c**) Completely weathered tuff exposed during excavation. (**d**) Construction of the overflow culvert buried under subgrade.

**Figure 11:**
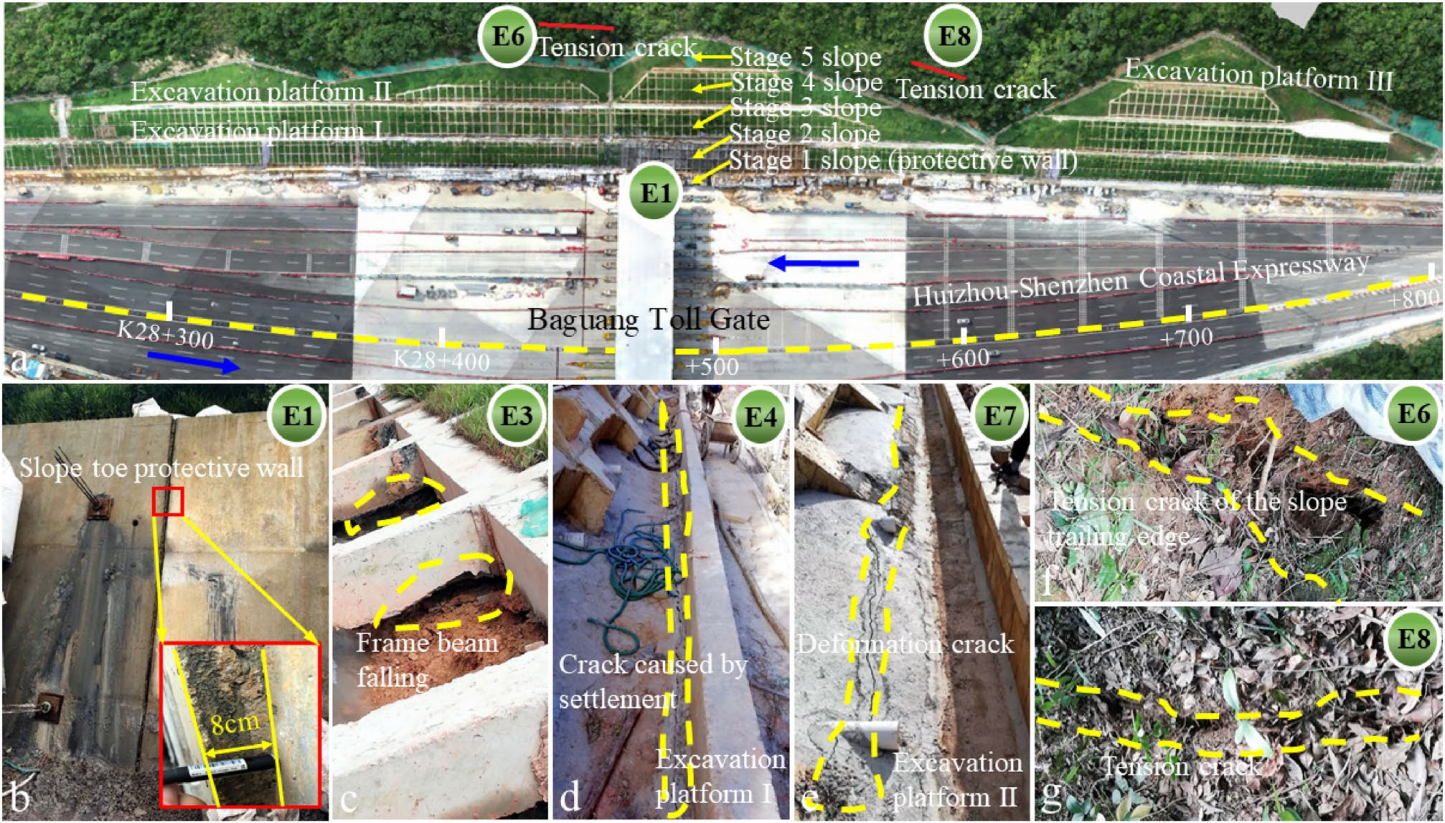
The panoramic photo of the supported slope, and the damage phenomenon in the deformation and failure evolution process (E1-E8 correspond to the time nodes in Fig. 9). (**a**) Supporting structure of slope; (**b**) deformation characteristics of the slope toe protective wall; (**c**) the first order frame beam deformed, damaged and out of ground contact; (**d,e**) drainage facilities cracking caused by the slope deformation; (**f,g**) tension cracks at the slope trailing edge.

After May 2018, extreme rainfall occurred continuously, with a large amount of rainwater penetrating the slope. Rainwater softened the underlying completely weathered tuff layer, resulting in the internal sliding surface. High-risk of sliding at any time poses a threat to the traffic safety. Typical events that trigger the slope gradual transition from stable state to critical sliding state are shown in Fig. 9. The events recorded in Fig. 9 are the results of the site investigation at different time nodes. Some observed conditions on site are shown in Figs. 10 and 11.

Figure 10 shows the soil excavation during construction. The main characteristics of shallow soil including: The fully weathered tuff layer had an obvious boundary with cohesive soil layer; The natural cohesive soil easy collapsed under the rainwater runoff action, resulting in collapse pit; Some boulders were mixed in cohesive soil, and the uneven distribution of boulders may affect the soil strength. There is a direct causal relationship between the complex structure of the soil layers and the sliding phenomenon of the reinforced slope under the extreme rainfall action.

Figure 11 shows the panoramic view after the construction completion, as well as the damage of different parts of the slope at different time nodes. There are five stage slopes and three excavation platforms after the slope support engineering finished (Fig. 11a, design parameters and construction distribution are shown in Fig. 3). Early observed slope deformation and failure occurred in the slope toe protective wall, and the anchored slope toe protective wall gradually produced large deformation under the strong thrust of the sliding mass (Fig. 11b). Then (Jun. 14, 2018), the first-order frame beam appeared deformation and suspension, which indicate that the slope deformation increased, and the slope stability deteriorated gradually (Fig. 11c). After 4 days, cracks appeared on the excavation platform I due to subsidence (Fig. 11d). After 40 days, deformation cracks were found on the excavation platform II (Fig. 11e). Tension cracks appeared one after another at the trailing edge of the slope (Fig. 11a,f,g). After the above deformation, the slope experienced two major storms, which directly brought the slope to the critical state of impending slip. High intensity emergency response measures were applied since September 2018. From the deformation phenomenon mentioned above, that the slope front edge damaged firstly, and the tension crack gradually appeared at the slope rear edge, this landslide can be regarded as atypical “Translational sliding” landslide^34^.

**Figure 12:**
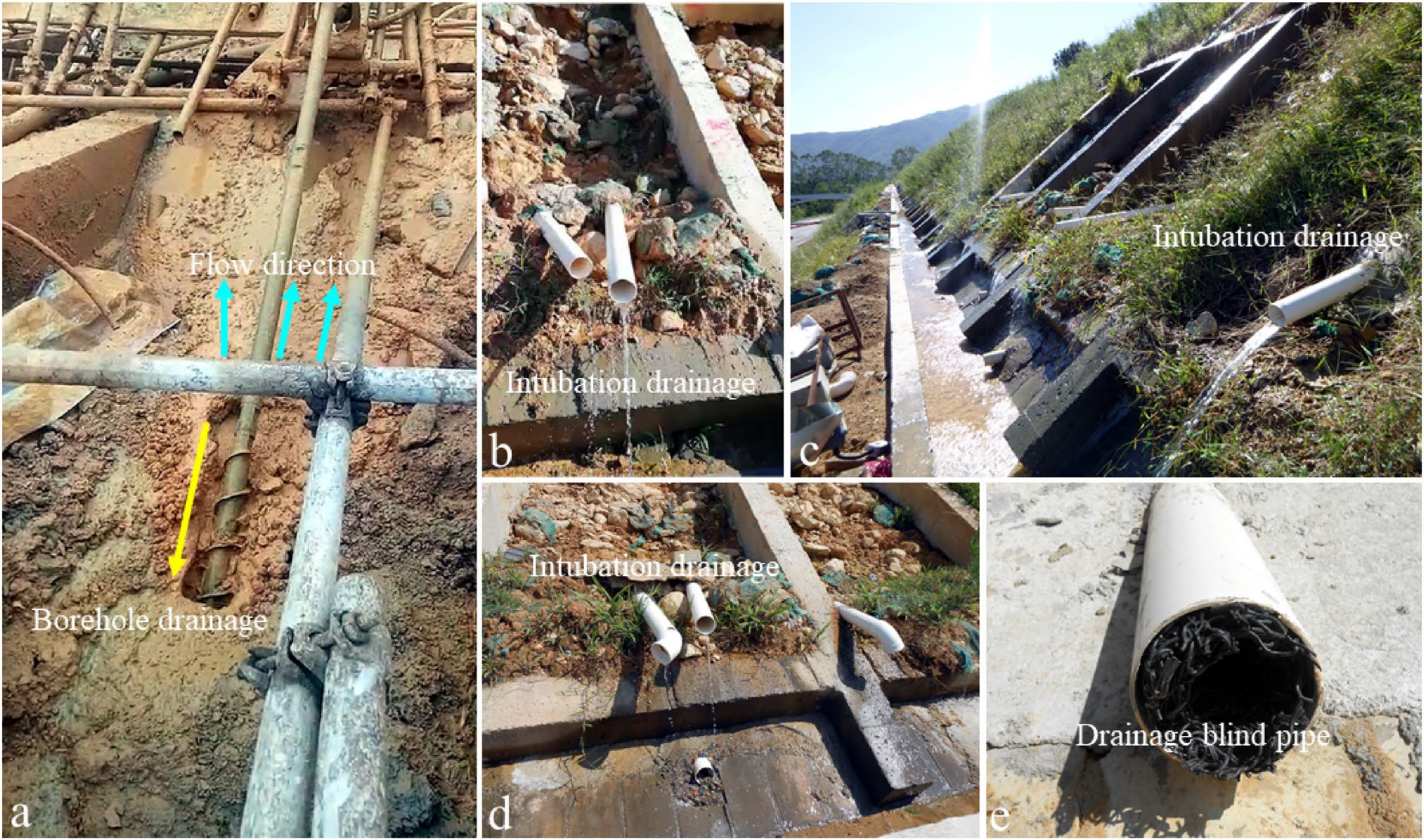
Drainage measures were taken to reduce landslide risk. (**a**) Borehole drainage (construction of deep horizontal drain hole); (**b–d**) slope insert tilted drainpipe; (**e**) buried drainage blind pipe.

Field observation also found that since August 2018, after heavy rainfall, the intercepting ditch was investigated and found that there was almost no water flowing into the intercepting ditch. This phenomenon indicates that a large amount of rainwater seeped into the slope and the surface runoff disappeared. Literature shows that this is the precursor of large-scale landslide^38,39^. To minimize the impact by the landslide, various drainage measures taken by the emergency response team are shown in Fig. 12. Artificial drilling and drainage were carried out at the slope seepage part (Fig. 12a). During the drilling process, the water flow in the slope would occasionally spray out, indicating that there was a large water pressure in the soil. After a heavy rainfall, a large amount of water could also be drained out using the inserted PVC pipe (Fig. 12b–d), indicating that a large amount of rainwater was contained in the slope soil. To drain the rainwater infiltrated into the soil in time, blind drainage pipes were directly buried in the soil during the emergency rescue process (Fig. 12e).

Another important aspect of site investigation is to determine the slope slip surface position based on borehole information. This is of great significance for landslide disaster prediction. Based on the distribution of boreholes in Fig. 6, the distribution position of slope slip surface is shown in Table 1. Table 1 shows that the maximum depth of the slip surface is 27 m (ZK19), and the slip surface depth at the slope toe is less than 9 m (CX-7, CX-1, CX-9). On the same cross sections (e.g., cross section 3-3, 4-4), with the increase of slope elevation, the slip surface depth gradually increases, indicating that the sliding boundary is outside the borehole investigation scope. Therefore, the landslide boundary in Fig. 6 is an estimated boundary (determined according to the tension crack location), and the actual landslide boundary may be larger.

**Table 1:** The slip surface depth relative to the ground obtained from some borehole positions (the borehole position is shown in Fig. 6).

Borehole no.	Slip surface depth (m)	Cross section no.	Borehole location
CX-7	6.5	K28 + 560 (3-3)	Protective wall top
ZK43	Less than 20	K28 + 560 (3-3)	Near anti-slide pile
CX-5	22.5	K28 + 560 (3-3)	Excavation platform I
CX-6	25.5	K28 + 560 (3-3)	Excavation platform II
ZK12	20.0	K28 + 560 (3-3)	Slope crest
CX-1	8.5	K28 + 610 (4-4)	Protective wall top
CX-12	25.5	K28 + 610 (4-4)	Excavation platform I
ZK19	27.0	K28 + 610 (4-4)	Excavation platform II
ZK21	21.0	K28 + 690 (5-5)	Slope crest
CX-9	7.5	K28 + 770 (6-6)	Protective wall top

### Monitoring results

#### Inclinometer results

**Figure 13:**
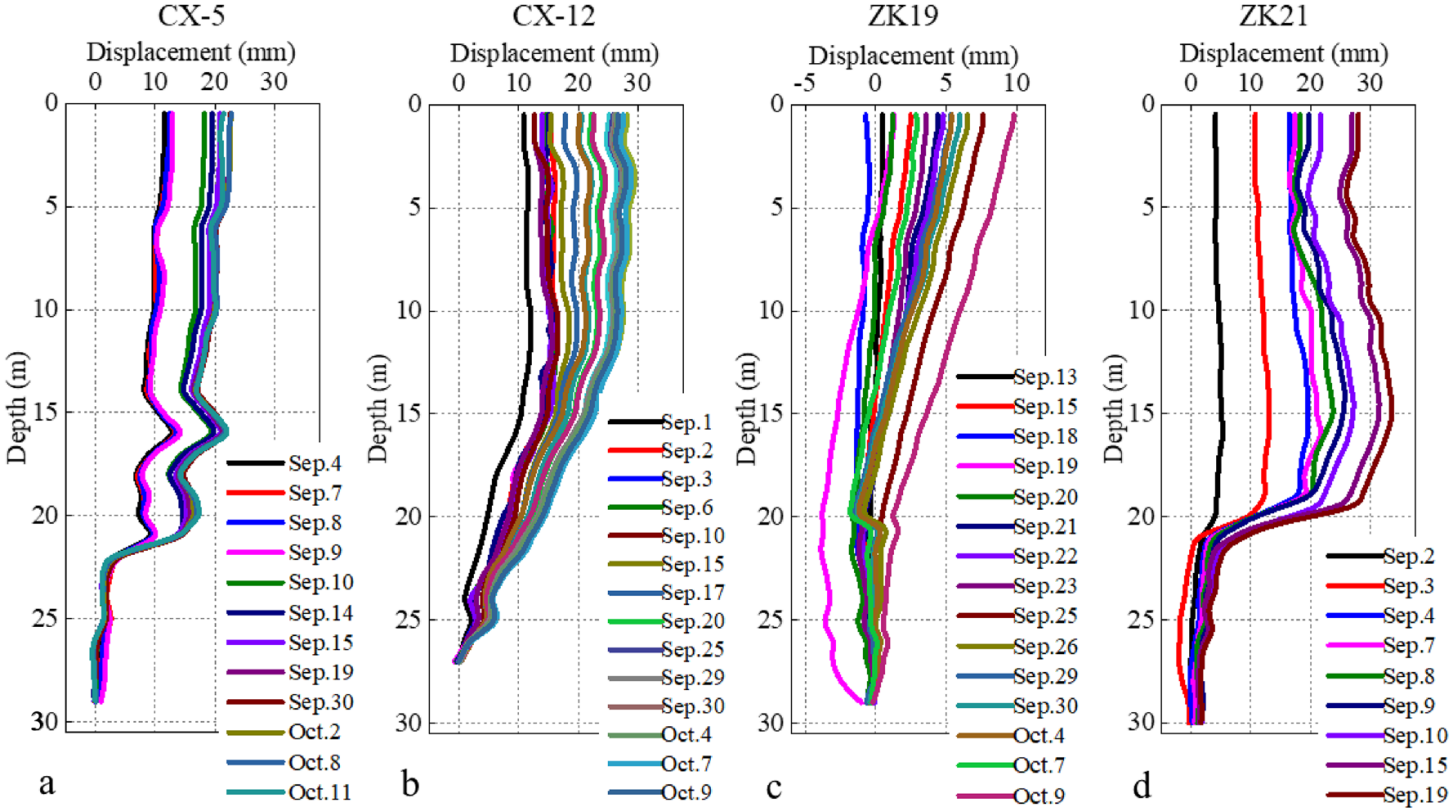
Inclinometer results of some boreholes after September 2018 (the borehole position is shown in Fig. 6).

Figure 13 shows the inclinometer results of some boreholes after September 2018. A positive displacement indicates that the deformation direction is the same as the sliding direction, and a negative displacement indicates that the deformation direction is opposite to the sliding direction. According to Fig. 9, the slope experienced heavy rainfall around September 1. Inclinometer results of borehole CX-5 (Fig. 9a) showed that the slip surface depth was about 21.5 m. There was “seesaw” in the depth range of 15 m to 22 m. There was an obvious “step” phenomenon in the inclinometer results during Sep. 9–10. The inclinometer results of borehole CX-12 (Fig. 9b) showed a convergence trend, the slip surface depth was about 27 m. It indicates that slope was in a “creep” state. Inclinometer results of borehole ZK19 (Fig. 9c) showed that the slip surface depth was about 28 m. At the depth of 20 m, there was an obvious “Z” type mutation of the displacement curve. The deformation curves at Sep. 9, Oct. 7 and Oct. 9 showed that the displacement curve at this borehole had obvious “oscillation” characteristics. Inclinometer results of borehole ZK21 (Fig. 9d) showed that the slip surface depth was about 20 m. Compared with the displacement at CX-5, CX-12 and ZK19 boreholes, ZK21 had no obvious gradual displacement change above the slip surface. This phenomenon indicates that the slope had slipped at ZK21 borehole location.

#### Slope toe protective wall deformation

**Figure 14:**
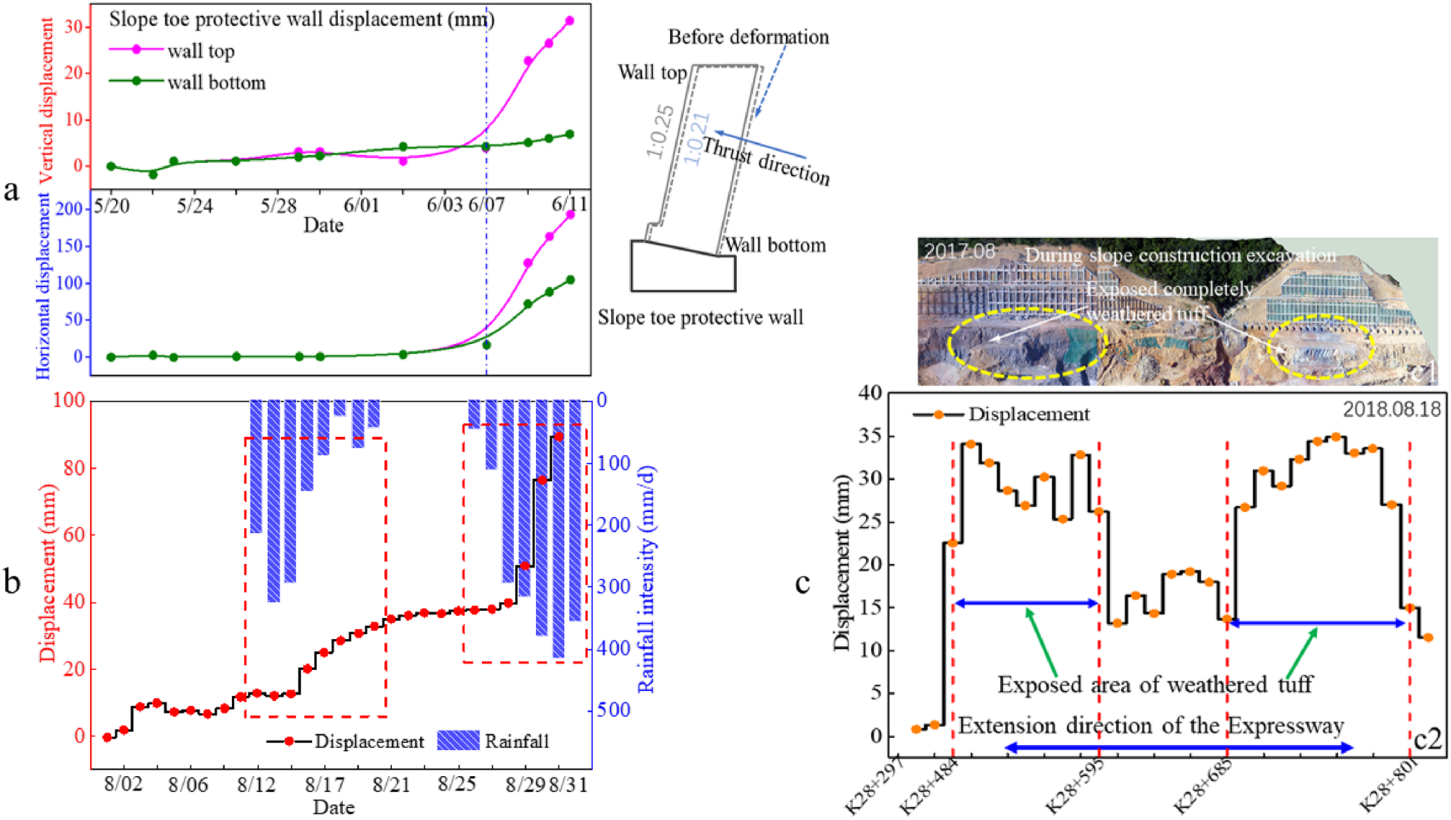
Typical deformation characteristics of slope toe protective wall: (**a**) location at K28 + 595 section; (**b**) (horizontal displacement) of slope toe protective wall (top) at K28 + 544 section after rainfall; (**c**) slope toe protective wall (top) deformation (horizontal displacement) in the extension direction of the Expressway on August 18, 2018, (c1) soil layer distribution during slope excavation, (c2) the displacement distribution.

Figure 14a shows the displacement variation of the slope toe protective wall (see Fig. 3) at K28 + 595 section in the horizontal and vertical directions, and the schematic diagram of stress and deformation ratio of the slope toe protective wall. Wall displacements showed that after June 7, 2018, the vertical displacement of the wall top increased significantly, and the horizontal displacement of the wall top and bottom also increased significantly. This phenomenon was directly related to the rainfall. The deformation and failure state of the protective wall was shown in Fig. 2c. In the slope slide evolution process, rainfall had obvious influence on the slope toe protective wall deformation. Figure 14b shows the influence of typical heavy rainfall on the slope toe protective wall deformation at the section K28 + 595. Each heavy rainfall could lead to a sharp increase in the displacement of the slope toe protective wall. The influence of rainfall on the slope toe protective wall deformation could gradually accumulate, and eventually led to the deformation aggravation and the slope stability deterioration.

Figure 14c shows the deformation characteristics of the slope toe protective wall along the expressway under the same observation standard. There were two areas with large deformation, namely K28 + 484 to K28 + 595 and K28 + 685 to K28 + 801. These two areas were also exposed area of the completely weathered tuff. This shows that rainfall infiltration has an obvious weakening effect on the soil strength in the completely weathered tuff distribution area. The water softening effect on the completely weathered tuff soil is also one of the fundamental reasons for the slope deformation and sliding. In addition, obvious delay phenomenon about the influence of the completely weathered tuff soil physical properties (water on its strength parameters) on the slope stability could be found clearly.

#### Anti-slide pile deformation

**Figure 15:**
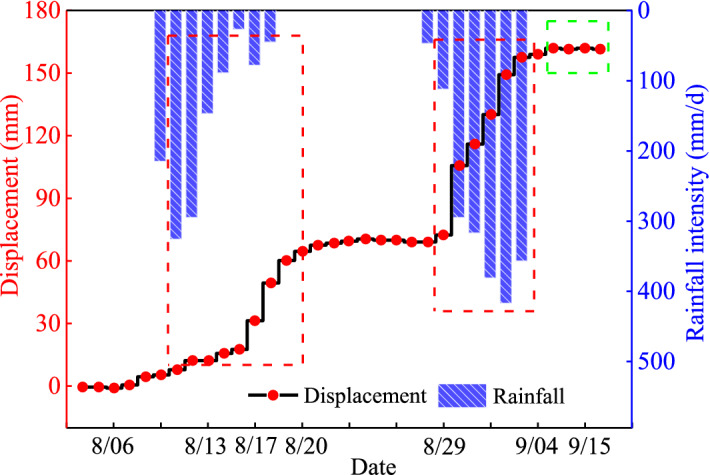
Displacement of anti-slide pile top (excavation platform I, near the section K28 + 426) during and after rainfall.

The deformation of the anti-slide pile (see Fig. 3) caused by rainfall is illustrated in Fig. 15. There was a rainfall started around August 10 and lasted nearly a week. The anti-slide pile showed obvious deformation of accelerating and tilting outwards at the end of the rainfall. The deformation of the anti-sliding pile had a delay relative to the rainfall process. Around August 30, the slope also experienced a heavy rainfall. However, the anti-slide pile had obvious deformation at the beginning of the rainfall. The deformation of the anti-slide pile continued but decreased gradually during the rainfall process. These phenomena show that the effect of rainfall on slope deformation had a cumulative effect. After the slope deformation develops to a certain extent, the rainfall event has a high probability to directly trigger the slope sliding. After September 15, the deformation of the anti-slide pile stopped (green frame line in Fig. 15), mainly due to the support measures taken by the emergency team.

#### Anchor cable tension

**Figure 16:**
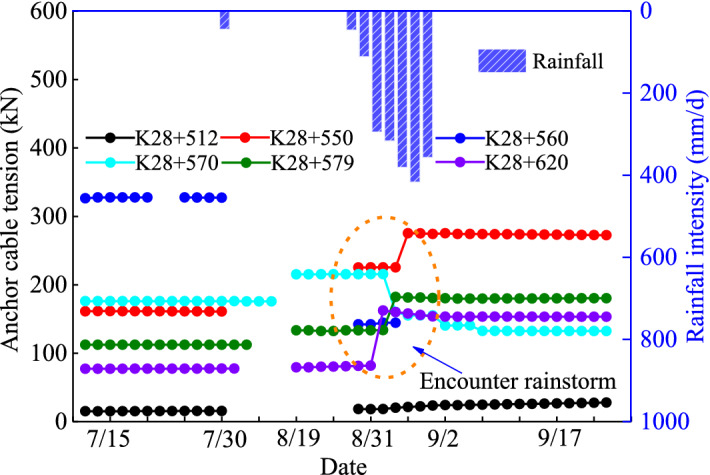
Changes of anchor cable tension at different positions before and after rainstorm.

The distribution of anchor cables in a typical slope profile is shown in Fig. 3. A certain density of anchor cables is distributed in the slope of the excavation platform I and above. Anchor cables are only installed in part of the slope with large excavation areas. Figure 16 shows the tension variation with time of some anchor cables in the slope at different positions along the extension direction of the expressway. The curve discontinuity is due to data acquisition failure in some time periods when emergency support was adopted for the slope safety. Between August 31 and September 2, 2018, heavy rainfall occurred in the slope area due to typhoon. Some anchor cables tension had changed significantly. Among them, anchor cable tension at K28 + 550, K28 + 579 and K28 + 620 sections increased significantly, and the anchor cable tension at K28 + 570 section decreased significantly. This phenomenon proves the adverse effect of heavy rainfall on slope stability. The variation difference of anchor cable tension reflects that the slope is not overall sliding.

#### Groundwater level change

**Figure 17:**
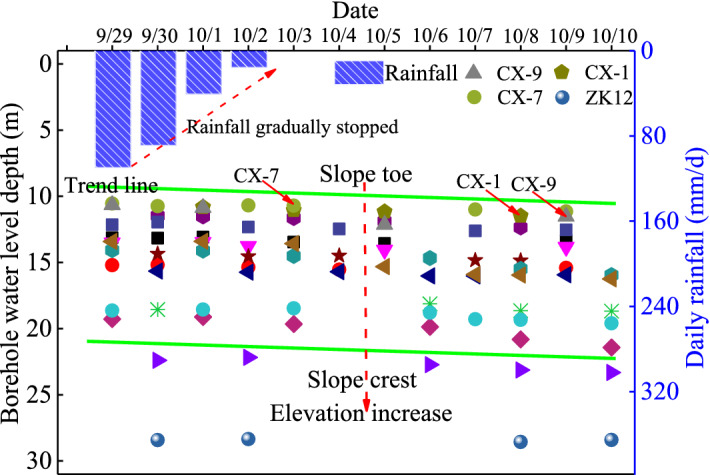
Groundwater level (relative to the ground at the borehole location) change in boreholes after rainfall. (Borehole location is shown in Fig. 6).

The groundwater level in a borehole is an important field monitoring index. The change of groundwater level in boreholes before and after rainfall can affect the slope stability directly, and its change after the rainfall reflects the comprehensive permeability of the soil layer. Figure 17 shows the groundwater level change with time in some boreholes on the site. As the rainfall gradually ceasing, the groundwater level in the borehole slowly decreased within 2 weeks. The groundwater level change caused by the rainfall process showed a fluctuating trend. This phenomenon was closely related to the geological conditions at the landslide location. The relative groundwater level of boreholes at the slope toe was low. With the increase of elevation, the relative groundwater level of boreholes gradually increases. The groundwater level in the landslide region was in the range of 10 to 30 m below the ground.

### Laboratory test results

Laboratory tests on the soil and rock samples obtained from field drilling were conducted to measure the physical and mechanical parameters. The test results are shown in Table 2. For the boulder with cohesive soil layer, there was a significant difference in shear strength parameters (cohesion, internal friction angle) between the sliding mass and sliding bed, and the shear strength of the sliding mass was significantly higher than that of sliding bed. The same situation also existed in the completely weathered tuff soil layer. In addition, the shear strength parameters of different soil layers decreased significantly after the soil was saturated, which also proved the negative impact of rainfall infiltration on slope stability. The strength of weathered tuff decreased significantly after soaking. Through the strength test of the sliding zone soil (the main sliding section and the anti-sliding section), the strength parameters of the soil were as follows: the cohesion was 9 kPa and the internal friction angle was 19.5°, which was significantly lower than the strength parameters of soil layers. The permeability coefficient of soil layer was obtained based on the constant head water injection test in field. With the increase of soil depth, the permeability coefficient decreased gradually. The low permeability of weathered tuff made it possible for the infiltration rainwater to stay in the soil layer, which provided the conditions for the formation of slip surface during the rainwater infiltration.

**Table 2:** Physical and mechanical parameters of rock and soil layer.

Stratum code and name		Natural bulk density *γ*(kN/m^3^)	Saturated bulk density *γ´*(kN/m^3^)	Shear strength				Permeability coefficient *K* (m/s)
				Cohesion *c* (kPa)		Internal friction angle *φ* (°)		
				Natural	Saturated	Natural	Saturated	
Q^ml^	Artificial fill	18.8	19.1	–	–	–	–	–
Q^pl+dl^	Boulder with cohesive soil (sliding mass)	19.0	19.5	18	15	20	16	3.56 × 10^–7^
Q^pl+dl^	Boulder with cohesive soil (sliding bed)	19.0	19.5	24	19	25	20	3.56 × 10^–7^
J_2-3_w	Completely weathered tuff (sliding mass)	19.0	19.5	11	9	25	20	2.5 × 10^–8^
J_2-3_w	Completely weathered tuff (sliding bed)	19.0	19.5	17	12	26	21	2.5 × 10^–8^
J_2-3_w	Strongly weathered tuff	19.5	20.0	28	23	32	28	–

### Theoretical calculation results

**Figure 18:**
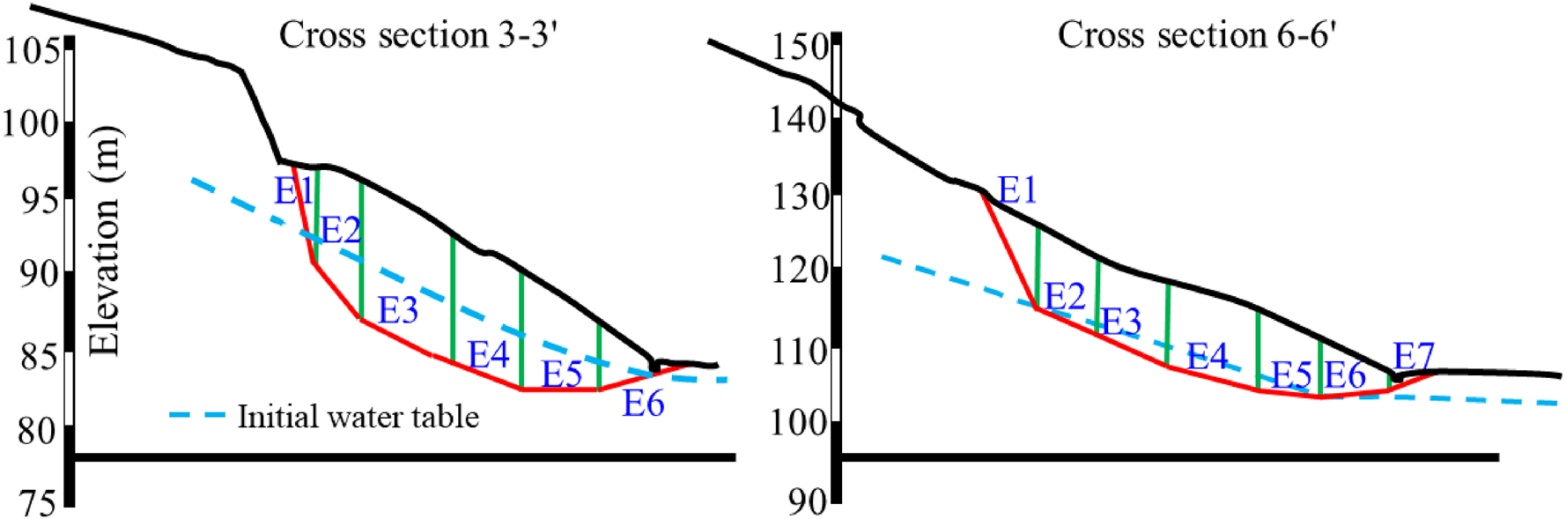
Schematic diagram of soil strip division in cross section 3-3′ and 6-6′.

To obtain the results on slope stability affected by the rainfall, the unbalanced thrust transfer coefficient method is used to calculate the two working conditions, i.e., without considering rainfall and considering continuous rainfall (rainfall intensity is 300 mm/day). Cross section 3-3′ and 6-6′ in Fig. 6 were selected for calculation. The sliding body was divided into strips. The division of soil strips and the distribution of initial groundwater level are shown in Fig. 18. Sliding body at cross section 3-3′ was divided into 6 strips, i.e., E1 to E6. Sliding body at the cross Sect.  6-6′ was divided into 7 strips, i.e., E1 to E7. The calculation results are shown in Tables 3, 4, 5 and 6.

**Table 3:** Calculation results of the slope stability in cross section 3-3′ (without considering rainfall).

No.	$\gamma$	$\gamma_{sat}$	$W_{i}$	$\theta_{i}$	$c_{i}$	$\varphi_{i}$	$\alpha_{i}$	$P_{Wi}$	$T_{i}$	$R_{i}$	$\psi_{i}$	*FS*
E1	18.50	19.00	6713.15	36.00	9.00	19.50	5.00	150.69	4075.06	2206.32	0.781	0.541
E2	18.50	19.00	11,192.50	20.00	9.00	19.50	23.00	1546.90	5372.85	3930.68	0.939	0.661
E3	18.50	19.00	14,010.45	14.00	9.00	19.50	23.00	1521.12	4891.82	5016.82	0.908	0.799
E4	18.50	19.00	7057.80	4.00	9.00	19.50	29.00	753.88	1175.57	2537.89	0.918	0.923
E5	18.50	19.00	2862.95	− 6.00	9.00	19.50	10.00	80.57	− 221.81	1107.51	0.623	1.036
E6	18.50	19.00	1089.45	− 41.00	9.00	19.50	10.00	18.58	− 703.05	372.45	0.948	1.134

See “Theoretical calculation” section for the meaning and unit of each parameter.

**Table 4:** Calculation results of the slope stability in cross section 3-3′ (considering continuous rainfall).

No.	$\gamma$	$\gamma_{sat}$	$W_{i}$	$\theta_{i}$	$c_{i}$	$\varphi_{i}$	$\alpha_{i}$	$P_{Wi}$	$T_{i}$	$R_{i}$	$\psi_{i}$	*FS*
E1	18.50	19.00	6805.80	36.00	9.00	19.50	5.00	312.19	4267.95	2262.32	0.777	0.530
E2	18.50	19.00	11,291.70	20.00	9.00	19.50	23.00	2322.12	6180.92	3949.32	0.933	0.601
E3	18.50	19.00	14,189.20	14.00	9.00	19.50	23.00	2917.98	6314.73	5000.86	0.894	0.680
E4	18.50	19.00	7168.70	4.00	9.00	19.50	29.00	1829.19	2157.87	2416.14	0.902	0.741
E5	18.50	19.00	2916.50	− 6.00	9.00	19.50	10.00	266.55	− 48.63	1108.21	0.572	0.822
E6	18.50	19.00	1113.40	− 41.00	9.00	19.50	10.00	101.76	− 666.42	355.96	1.001	0.943

**Table 5:** Calculation results of the slope stability in cross section 6-6′ (without considering rainfall).

No.	$\gamma$	$\gamma_{sat}$	$W_{i}$	$\theta_{i}$	$c_{i}$	$\varphi_{i}$	$\alpha_{i}$	$P_{Wi}$	$T_{i}$	$R_{i}$	$\psi_{i}$	***FS***
E1	18.50	19.00	3935.60	45.00	9.00	19.50	21.00	110.74	2884.05	1180.52	0.644	0.409
E2	18.50	19.00	7895.40	25.00	9.00	19.50	20.00	541.42	3876.10	2716.26	0.945	0.606
E3	18.50	19.00	11,646.65	20.00	9.00	19.50	20.00	955.26	4938.65	4109.57	0.963	0.714
E4	18.50	19.00	14,571.10	16.00	9.00	19.50	17.00	933.25	4949.45	5246.74	0.878	0.829
E5	18.50	19.00	6175.30	3.00	9.00	19.50	17.00	465.16	774.54	2334.74	0.891	0.951
E6	18.50	19.00	1091.50	− 10.00	9.00	19.50	17.00	64.91	− 131.70	423.31	0.756	0.996
E7	18.50	19.00	773.35	− 35.00	9.00	19.50	17.00	11.11	− 436.74	313.93	1.007	1.081

See “Theoretical calculation” section for the meaning and unit of each parameter.

**Table 6:** Calculation results of slope stability in cross section 6-6′ (considering continuous rainfall).

No.	$\gamma$	$\gamma_{sat}$	$W_{i}$	$\theta_{i}$	$c_{i}$	$\varphi_{i}$	$\alpha_{i}$	$P_{Wi}$	$T_{i}$	$R_{i}$	$\psi_{i}$	***FS***
E1	18.50	19.00	4026.10	45.00	9.00	19.50	21.00	759.38	3540.61	1296.61	0.609	0.366
E2	18.50	19.00	8027.50	25.00	9.00	19.50	20.00	1445.04	4832.10	2786.55	0.936	0.512
E3	18.50	19.00	11,818.00	20.00	9.00	19.50	20.00	2127.37	6169.36	4166.59	0.956	0.591
E4	18.50	19.00	14,801.00	16.00	9.00	19.50	17.00	2277.58	6356.94	5316.69	0.856	0.675
E5	18.50	19.00	6260.50	3.00	9.00	19.50	17.00	963.36	1262.40	2322.19	0.870	0.761
E6	18.50	19.00	1109.60	− 10.00	9.00	19.50	17.00	170.75	− 40.55	412.61	0.717	0.791
E7	18.50	19.00	792.30	− 35.00	9.00	19.50	17.00	121.92	− 379.38	288.51	1.059	0.849

Table 3 shows the calculation parameters and results of each strip of cross section 3-3′ without considering rainfall. It is assumed that the natural bulk density, saturated bulk density, cohesion and internal friction angle of each strip are the same in the calculation. The interaction force between strips is gradually accumulated from E1 to E6 through the transfer coefficient. From the calculated results, it can be seen that only the FS of E5 and E6 is greater than 1, and the FS of E6 is greater than 1, which indicates that the slope is generally in stable state. Table 4 shows the scenario considering transient continuous rainfall (rainfall intensity is 300 mm/day). Calculation parameters are kept the same as the scenario without considering rainfall. Rainfall can cause obvious changes in the strip weight, the osmotic pressure, the anti-sliding force, the sliding force, and the reaction force, which are reflected in the calculated FS. Compared with the FS obtained in Table 3, the FS of the same strip in Table 4 is significantly reduced. The FS of E6 strip is 0.943, indicating that landslide has happened.

The calculation parameters and results of each strip of cross section 6-6′ without considering rainfall and considering transient continuous rainfall (rainfall intensity is 300 mm/day) were displayed in Tables 5 and 6 respectively. Similar to the scenario of cross section 3-3′, transient continuous rainfall will significantly reduce the FS of each strip. For the case of without considering rainfall, the FS of strip E7 is 1.081, while considering rainfall, the FS of E7 decreases to 0.849. If the influence of rainfall infiltration on the shear strength parameters of rock and soil is considered, the influence of rainfall events on the FS of each strip will be more significant. Therefore, rainfall infiltration plays an important role in this landslide event.

## Discussion

### Evolution characteristics

The sliding characteristics (such as deformation cracks) in landslide starting stage are generally an important index to identify the slope sliding risk^24,40^. However, before the observable large cracks appearing, the slope deformation can be observed firstly on the supporting structures. The events on the timeline in Fig. 9 show that the landslide initially deforms from the slope toe and gradually extends to the slope crest. When the slope deformation lasts for several months later, large deformation cracks finally appear at the slope trailing edge. This is a typical step-by-step deformation feature from bottom to top. One of its typical features is progressive failure^41^, which may provide response time for the emergency disposal in case of this disaster.

The runoff generated by rainfall, on the one hand, can increase the soil bulk density and reduce the soil strength; on the other hand, the runoff (accumulated flow) cannot be formed on the slope surface during heavy rainfall indicates the landslide risk increase^42^. This landslide has many man-made drainage ditches, which work normally before the slope deformation. When the slope deformation lasts for a period, however, there is no ponding on the slope and drainage ditch during heavy rainfall. Large amount of rainwater flowing into the slope directly suggests a clear risk signal^38^. To reduce the risk, special drainage measures were taken as shown in Fig. 10. The accumulated water in the slope was released by horizontal drilling holes (Fig. 12a) or inserted drainage pipes (Fig. 12b–d), so that the water can flow out of the slope as soon as possible. In the drilling and drainage process, water jet phenomenon indicates that there was a large hydrostatic pressure in the slope. The temporary drainage measures can effectively alleviate the damage of water pressure in soil on the supporting structures. In addition, there is a close relationship between rainwater infiltration quantity and slope stability.

Determination of the slope sliding surface is an important preliminary work to evaluate landslide size and impact^43^. According to the position data of the slope slip surface obtained from field drilling (Table 1), taking the borehole on the cross section 3-3′ as an example, the depth of the slip surface at CX-7, ZK43 and ZK12 is shallower than that at CX-5 and CX-6, which indicates that the slip surface tends to be arc-shaped (the middle part is deep, and the two ends are relatively shallow). This is very close to the shape of slope slip surface in theory^44^. Thus, to investigate the distribution of slip surface, the exploration efficiency can be optimized by reasonably arranging the drilling position.

The influence of extreme rainfall on the slope support structures (slope toe protective wall, anti-slide pile and anchor cable) is extremely significant. Figure 14a,b show that rainfall events can cause obvious accelerated deformation of slope toe protective wall. Figure 14c shows that the slope deformation along the extension direction of the expressway shows obvious differentiation, which is closely related to the distribution of rock and soil layers in the slope, mainly due to the obvious strength reduction of the completely weathered tuff soil after encountering the infiltrated rainwater. The changes of the anti-slide pile displacement (Fig. 15) and the anchor cable tension (Fig. 16) show that the action load on the slope support structure can surge during heavy rainfall. Such significant change of the action load is usually the direct cause for triggering landslide^45^. Based on the significant change of the action load on slope support structure during heavy rainfall, it can be judged that extreme rainfall is likely to become the direct factor triggering large-scale landslides.

### Sliding mechanism analysis

During the construction, slope cutting is required on the roadside of the expressway, and the maximum slope cutting height is up to 32 m, which becomes not only the early trigger factor, but also the root cause of the landslide. Engineering excavation decreases the stability of the original soil layer, resulting in settlement and deformation. The deformation further leads to tension cracks in the mountain at the slope back. Four months after the completion of slope construction, the slope region suffered from continuously extreme rainfall. A large amount of rainwater collected on the mountain surface and accelerated infiltration into the slope through the tension cracks.

After rainwater infiltrates into the slope, it affects the slope stability in several ways, including: (1) rainwater infiltration leads to the weight increase of the soil mass, which further leads to the anti-sliding force decrease; (2) The osmotic pressure increase leads to the decrease of slope stability, which directly causes the stress increase of the slope protection facilities. Tables 3, 4, 5 and 6 show that the FS of cross section 3-3′ and cross section 6-6′ were significantly reduced after suffering from continuous rainfall. The main reason is that the infiltrated rainwater changes the weight, the osmotic pressure, the anti-sliding force, and the sliding force of the sliding mass^46,47^. Besides, in the Baguang Toll Landslide, the rainwater can soften the fully weathered tuff soil and reduce its strength, which induces the deformation of the completely weathered tuff distribution zone more significantly (Fig. 14c). This important factor has not been fully considered in the slope construction design. The rainy season was avoided in the whole process of excavation and slope support construction, resulting in the failure to accurately evaluate the slope deformation risk caused by rainwater infiltration.

**Figure 19:**
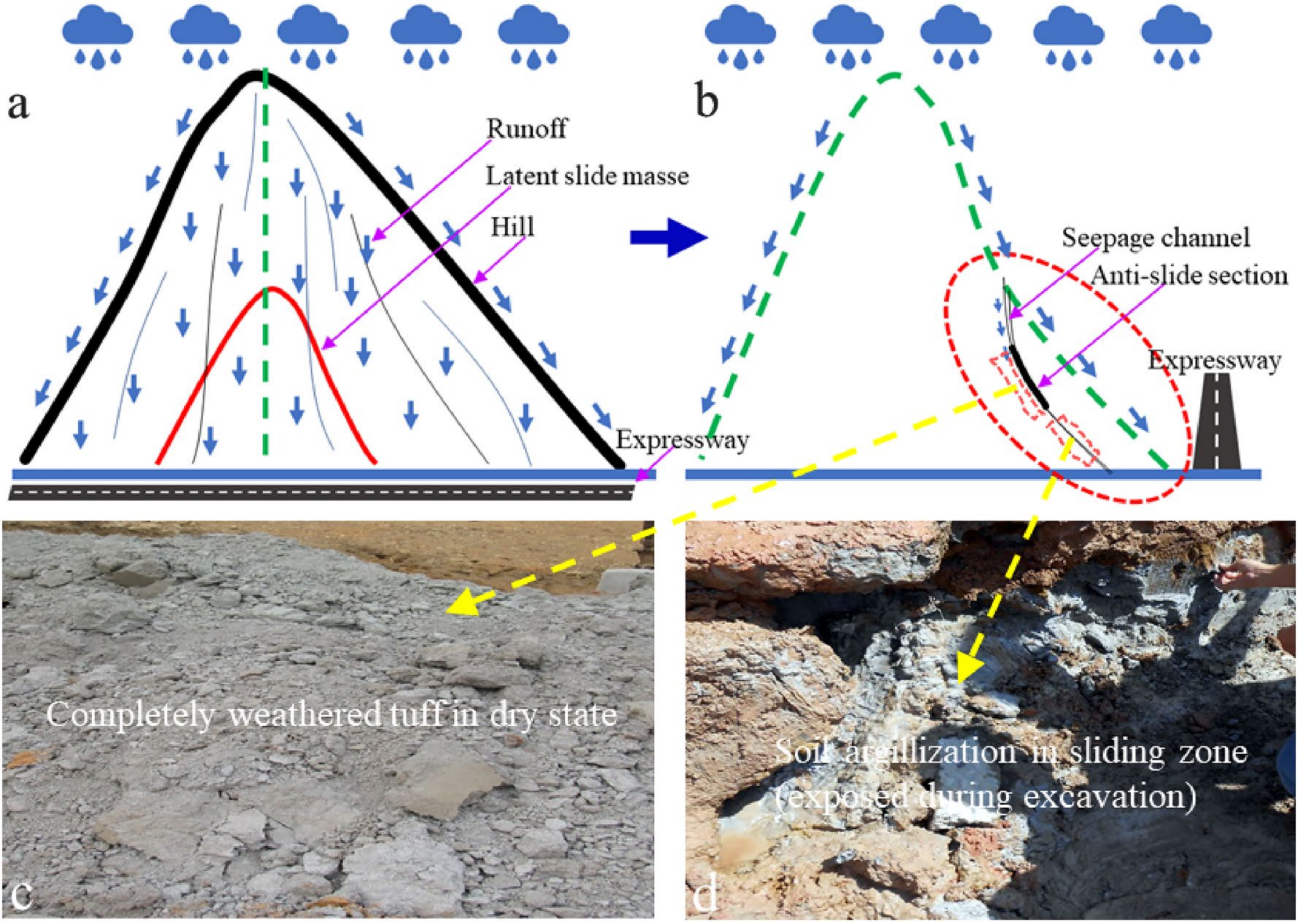
Occurrence mechanism of slope sliding caused by rainwater infiltration. (**a**) Schematic diagram of the sliding mass; (**b**) profile of the sliding mass (lateral view); (**c**) completely weathered tuff in natural dry state; (**d**) mudding state of the slip zone soil mixed by completely weathered tuff and silty clay.

**Figure 20:**
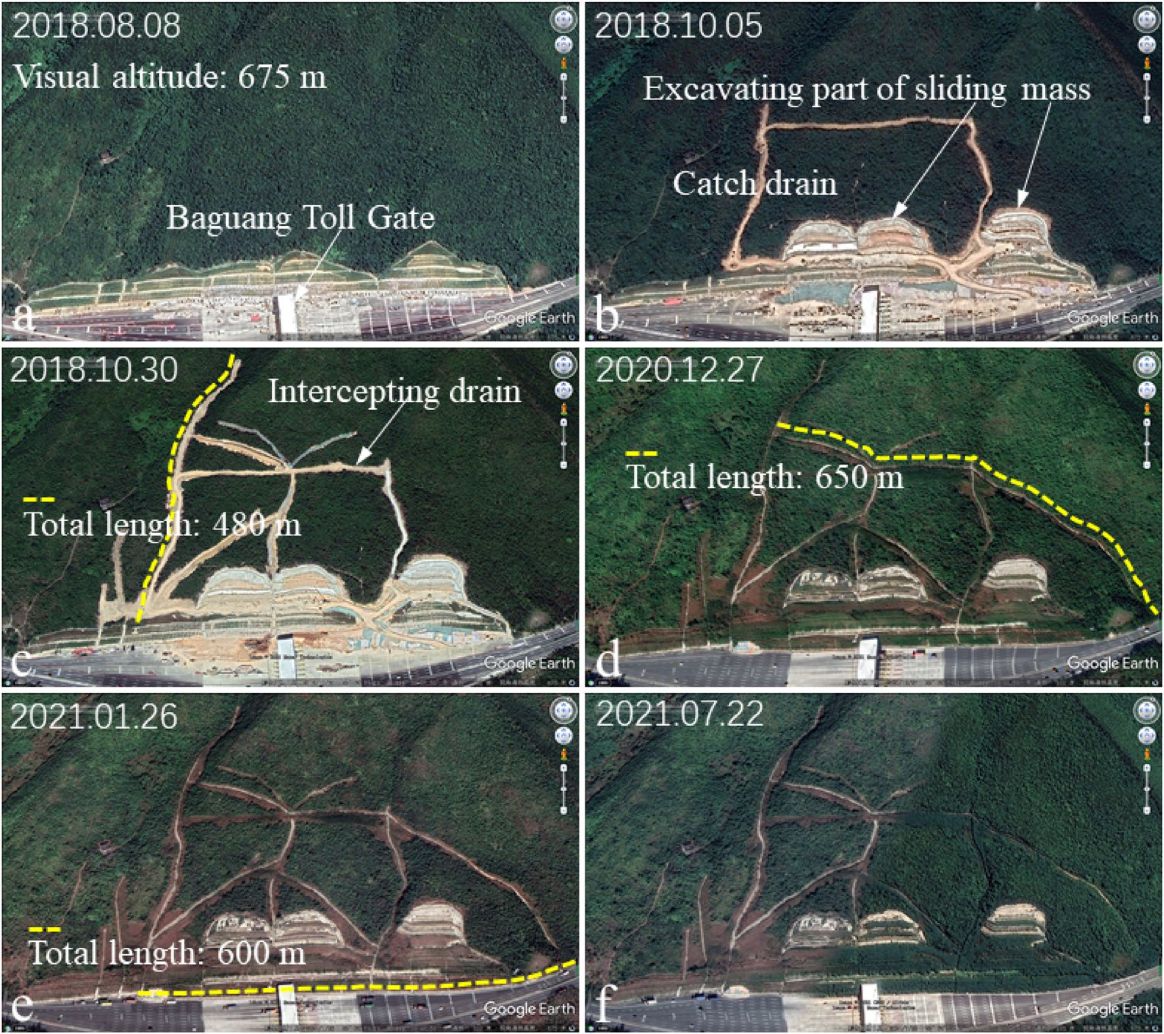
Setting and current situation of intercepting ditches and drainage ditches that block the runoff on slope surface at different time nodes. (**a**) The slope in a critical sliding state after extreme rainfall; (**b,c**) drainage ditch and intercepting ditch set on the mountain above the slope top; (**d**) excavation area covered by vegetation gradually; (**e,f**) current status of the Baguang Toll Landslide.

Figure 19 shows the occurrence mechanism of slope sliding triggered by rainfall. A key factor is the seepage channel. Preventing rainwater infiltration from seepage channel is the fundamental measure to eliminate the large-scale sliding risk. To achieve this goal, emergency team tries to find the seepage channel in the direction of the mountain top and prevent the runoff from flowing into the slope using intercepting and drainage ditches (Fig. 20). Figure 20 also shows the working path of seeking the seepage channel at different time node. To eliminate the large-scale sliding risk, the emergency team finally adopted the treatment of excavating part of the sliding mass and intercepting the runoff (Fig. 20b,c).

## Conclusions

This study analyzes the evolution process and failure mechanism of a large expressway roadside landslide. Based on the data collected on site, the deformation and evolution characteristics, triggering factors at the key time nodes in the slope start-up and sliding process are analyzed to reveal the slope start-up characteristic and sliding mechanism. The analysis results can help to the early identification and control of the similar landslide. The main conclusions can be drawn as following:

1. The mountain excavation in expressway construction is the fundamental reason for the occurrence of the landslide. The initial deformation and failure occurred on the slope toe protective wall. The deformation and failure gradually developed from the slope toe to the top, and finally led to tensile cracks at the slope trailing edge.
2. The on-site emergency disposal measures including borehole drainage, slope insert tiled drainage pipe, burning blind drainage pipes can be used to reduce landslide risk, and the effect is obvious. Extremely heavy rainfall can significantly accelerate deformation of slope toe protective wall. Anti-slide pile and anchor cable tension can be used as an important parameter for risk identification.
3. Engineering excavation deceases the stability of the original soil layer, resulting in the slope deformation. Deformation further leads to tension cracks, which become the seepage channel of rainwater. The decrease of the fully weathered tuff soil strength and the increase of hydrodynamic pressure in soil caused by rainwater infiltration are responsible for the slope creep and the final landslide.

## Data Availability

All data generated or analyzed during this study are included in this published article.
